# Reliability of movement control tests in the lumbar spine

**DOI:** 10.1186/1471-2474-8-90

**Published:** 2007-09-12

**Authors:** Hannu Luomajoki, Jan Kool, Eling D de Bruin, Olavi Airaksinen

**Affiliations:** 1 Physiotherapie Reinach, 5734 Reinach, Switzerland; 2 University of Kuopio, Kuopio, Finland; 3 Institute of Physiotherapy, Department of Health, Zürich University of Applied Sciences, Winterthur, Switzerland; 4 Department of Rheumatology and Institute of Physical Medicine, University Hospital Zürich, Switzerland; 5 Institute of Human Movement Sciences and Sport, ETH Zürich, Switzerland; 6 Department of Physical and Rehabilitation Medicine, University Hospital of Kuopio, Finland

## Abstract

**Background:**

Movement control dysfunction [MCD] reduces active control of movements. Patients with MCD might form an important subgroup among patients with non specific low back pain. The diagnosis is based on the observation of active movements. Although widely used clinically, only a few studies have been performed to determine the test reliability. The aim of this study was to determine the inter- and intra-observer reliability of movement control dysfunction tests of the lumbar spine.

**Methods:**

We videoed patients performing a standardized test battery consisting of 10 active movement tests for motor control in 27 patients with non specific low back pain and 13 patients with other diagnoses but without back pain. Four physiotherapists independently rated test performances as correct or incorrect per observation, blinded to all other patient information and to each other. The study was conducted in a private physiotherapy outpatient practice in Reinach, Switzerland. Kappa coefficients, percentage agreements and confidence intervals for inter- and intra-rater results were calculated.

**Results:**

The kappa values for inter-tester reliability ranged between 0.24 – 0.71. Six tests out of ten showed a substantial reliability [k > 0.6]. Intra-tester reliability was between 0.51 – 0.96, all tests but one showed substantial reliability [k > 0.6].

**Conclusion:**

Physiotherapists were able to reliably rate most of the tests in this series of motor control tasks as being performed correctly or not, by viewing films of patients with and without back pain performing the task.

## Background

Low back pain [LBP] is a huge social and financial problem for all western societies [1]. According to evidence based guidelines [2] up to 90% of all LBP is classified as non specific low back pain [NSLBP]. This means that in a medical sense, the cause of the back pain is not clear. Although LBP classification systems have been proposed, it is still unclear which clinical tests can be reliably used to allow subgroup categorization. The identification of subgroups of LBP has been identified as a major future research topic [2]. Several authors suggest that because NSLBP is a benign problem, the emphasis should be on clinical tests and assessments [1-5].

An earlier systematic review of treatments used for NSLBP revealed that few studies had addressed the issue of clinical subgroups [6]. Widely used synonyms for the movement control dysfunctions are movement impairment syndromes, relative flexibility [7] motor control dysfunctions [4,8,9] and movement dysfunctions [10,11]. In this publication we will use the term movement control dysfunction [MCD] which is diagnosed based on the observation of active movements.

One of the common features of MCD is a reduced control of active movement. These patients might form an important subgroup of patients with non specific low back pain. The assumption underlying MCD is that due to impaired control of the active movements of the back, people may be damaging themselves by inadvertently moving in a provocative manner. Instead of pain avoiders, O'Sullivan describes these back pain patients as pain provocateurs [4]. Relative flexibility theory [7,12] suggests that movement occurs through the pathway of least effort eg. if the hip flexion is relatively stiff compared to the low back, then the flexion movement is more likely to happen in the back leading to a flexion related back pain problem.

Examination and treatment options for movement impairment dysfunctions have been proposed. [4,5,7,9-11,13,14]. However, only a few studies have been performed to determine test reliability. Outcome intervention studies using this subgroup are yet to be reported. Van Dillen [12,15] and her group examined the reliability of physical examination items used for classification of patients with low back pain. They examined 28 items [N = 138] and found overall reliability of symptom behaviour to be very good [kappa > 0.75]. The assessment of alignment of the spine was found to be moderate [k = 0.27–0.66], and good [k = 0.21–0.78] for most of the movement items. The authors stated that it was often difficult to attain good reliability by judgements made on visual and tactile information. However, they believed that with enough training on each test, there would be significant improvement in those judgements.

In a study by Dankaerts et al [16] two expert clinicians classified 35 NSLBP individuals on the basis of a subjective and physical examination. They found an almost perfect agreement [k = 0.96 and percentage agreement 97 %] between the two examiners. Then, 25 videos in conjunction with the pain history and behaviour of the cases were randomised and 13 additional clinicians classified the same cases. Kappa-coefficients [mean 0.61 and range 0.47–0.8] and % agreement [mean 70% and range 60–84%] indicated substantial reliability. They stated that increased familiarity with the classification system improved reliability. In this study, however,, individual tests were not identified, thus no conclusions pertaining to the reliability of the individual tests can be made.

Hicks et al [17] examined the reliability of clinical measures, such as active and passive movement testing, palpation and provocation tests, for the identification of lumbar segmental instability. They found good Kappa values [mean 0.60 and 95% confidence intervals 0.43–0.73] for the active movement observational tests, but poor values for segmental passive tests [range 0.02–0.26]. A better reliability [k range 0.25–0.55] was demonstrated for the passive pain provocation tests.

There is some evidence for better reliability for the active movement tests than for passive movement tests. Therefore a test battery for MCD of the low back was developed. The judgement of quality of movement relies on inspection and we wanted to study the reliability of this ability separated from all other information gained from subjective or objective assessments. The aim of this study was to determine the inter- and intra-observer reliability of MCD tests of the lumbar spine. Further on we wanted to know, whether the amount of experience has an effect on reliability.

## Methods

### Study design

An inter- and intra-observer reliability study was conducted. Patients were videoed in a standardized manner by performing a set of ten active movement tests. Four physiotherapists blinded to the patients and to each other rated the test performances as either correct or not correct. The study was approved by the Ethics committee of the health authorities of the government of Canton Aargau, Switzerland and it was carried out in compliance of Helsinki declaration of human research. Written informed consent was obtained from all patients.

### Study sample

The sample size requirement for comparing two coefficients of inter-observer agreement was calculated for dichotomous outcome variables [Donner's method [18] by selecting the level of significance as alpha = 0.01 and power [beta = 0.80] for testing Ho: k1 > 0.4 versus H1: k1 < 0.4, the required sample size for group testing would be 36 cases for good [k index > 0.40] strength of agreement [sample size calculation table by Sim & Wright [18]. The sample size was set as N = 40 to cater for a potential dropout rate of 10%. Table 1 shows the background data of the patients in the videos.

**Table 1:** Subject characteristics on the videos

	Patients without back pain	Patients with back pain	Total
N =	13	27	40
Female/Male	8/5	18/9	26/14
Mean age (years) SD	55.1 (5.1)	50.8 (6.2)	52.1 (5.5)
Roland Morris score (SD)		8.5 (5.5)	

Forty patients from a private physiotherapy practice [Reinach, Aargau, Switzerland] were asked to participate. The background and the aims of the study were explained and all patients signed a written informed consent. Twenty seven patients had non specific low back pain [NSLBP] and 13 were without LBP but were receiving treatment for other musculoskeletal problems. We considered it important to also include in the sample subjects who would be performing the tests very well in order to increase the variability in the test sample and, thus, avoid a possible bias of the results through too many incorrect test results. Exclusion criteria were serious pathologies such as non-healed fractures, anomalies, tumours and acute trauma. Patients with acute back pain were excluded as well, as the pain may have prevented them from accomplishing the tests. Patients had to be able to understand the instructions in German.

### Rating of test performance

Prior to rating the patient's test performance using the video recordings, the study conductor presented typical clinical patterns of MCDs and discussed the scoring criteria with the raters. Four physiotherapists watched the videos one time and independently rated test performance. Two raters were clinical specialists in the field with 25 years of working experience. Each had a post-graduate degree in manual therapy and was experienced with the assessment of MCD. The other two raters were physiotherapists with 5 years of working experience. Neither of them had a post-graduate degree. They participated in a three-day course of movement control dysfunctions given by the study conductor.

Raters were blinded to the diagnosis and to the results of the examination of the patients. Raters watched each video recording only once. For the analysis of intra-observer reliability, one person of each pair rated the same videos two weeks apart.

### Test protocol

All patients received standardized instructions. For example in the prone knee bend test the assignment was: "please bend your knee as far as you can without moving your back" and: "keep your back in neutral position, do not let it move while bending the leg", If the patient did not understand how to perform the test, it was explained again and demonstrated by the examiner. If the patient was still performing the test incorrectly, it was permitted. The order of the tests was standardized. Videos were prepared anonymously, without showing the face, or filmed from behind so that the person could not be identified. One person [HL] made all the videos and was not involved in test performance rating. Patients wore underwear so that posture and movements of entire spine, hips and lower extremities could be observed. Raters were blinded to each other and to the medical history of each subject.

**Figure 1:**
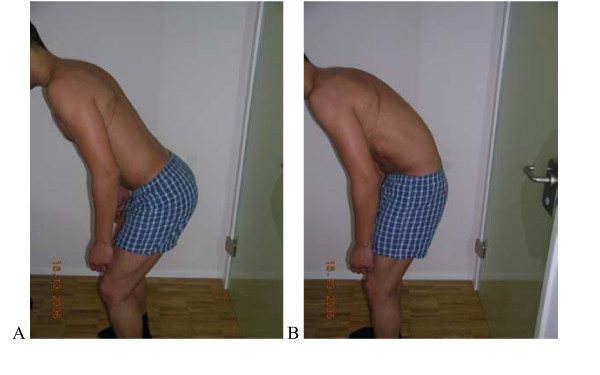
**Test protocol – "Waiters bow"**. Flexion of the hips in upright standing without movement (flexion) of the low back. **A**. **Correct -**Forward bending of the hips without movement of the low back (50–70° Flexion hips). **B Not correct **Angle hip Fx without low back movement less than 50° or Flexion occurring in the low back. **Rating protocol: **As patients did not know the tests, only clear movement dysfunction was rated as "not correct". If the movement control improved by instruction and correction, it was considered that it did not infer a relevant movement dysfunction.

**Figure 2:**
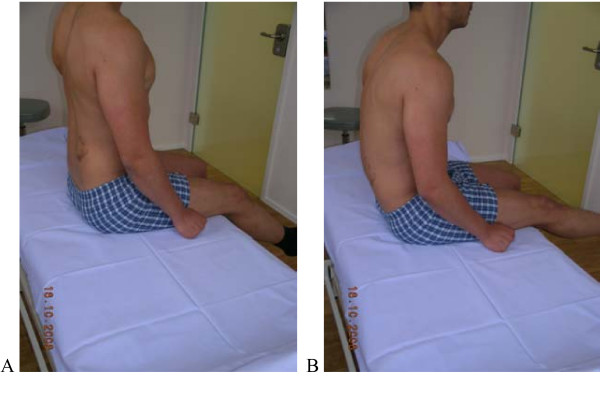
**Test protocol – Sitting knee extension**. Upright sitting with corrected lumbar lordosis; extension of the knee without movement (flexion) of low back **A**. **Correct – **Upright sitting with corrected lumbar lordosis; extension of the knee without movement of LB (30–50° Extension normal). **B Not correct **Low back moving in flexion. Patient is not aware of the movement of the back. **Rating protocol: **As patients did not know the tests, only clear movement dysfunction was rated as "not correct". If the movement control improved by instruction and correction, it was considered that it did not infer a relevant movement dysfunction.

**Figure 3:**
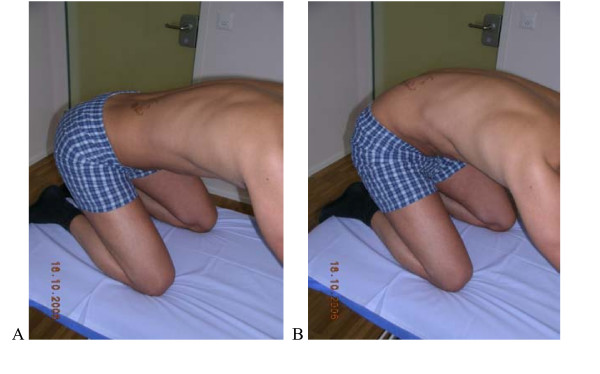
**Test protocol Rocking backwards**. Transfer of the pelvis backwards ("rocking") in a quadruped position keeping low back in neutral. **A**. **Correct -**120° of hip flexion without (Fx) movement of the low back by transferring pelvis backwards. **B Not correct **Hip flexion causes flexion in the lumbar spine (typically the patient not aware of this). **Rating protocol: **As patients did not know the tests, only clear movement dysfunction was rated as "not correct". If the movement control improved by instruction and correction, it was considered that it did not infer a relevant movement dysfunction.

**Figure 4:**
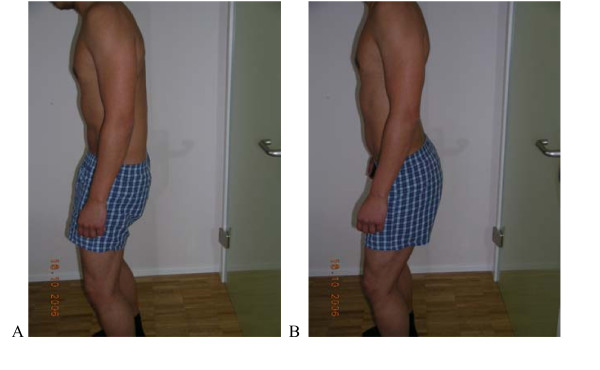
**Test protocol Dorsal tilt of pelvis**. Actively in upright standing. **Correct – **Actively in upright standing (Gluteus activity); keeping thoracic spine in neutral, lumbar spine moves towards Fx. **B Not correct **Pelvis doesn't tilt or low back moves towards Ext./No gluteal activity/compensatory Fx in Thx. **Rating protocol: **As patients did not know the tests, only clear movement dysfunction was rated as ''not correct''. If the movement control improved by instruction and correction, it was considered that it did not infer a relevant movement dysfunction.

**Figure 5:**
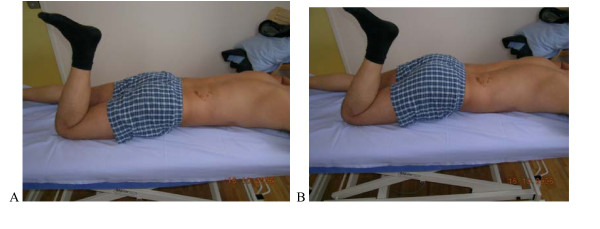
**Test protocol -Prone lying active knee Flexion. A**. **Correct – **Active knee flexion at least 90° without extension movement of the low back and pelvis. **B Not correct **By the knee flexion low back does not stay neutral maintained but moves in Ext. **Rating protocol: **As patients did not know the tests, only clear movement dysfunction was rated as "not correct". If the movement control improved by instruction and correction, it was considered that it did not infer a relevant movement dysfunction.

**Figure 6:**
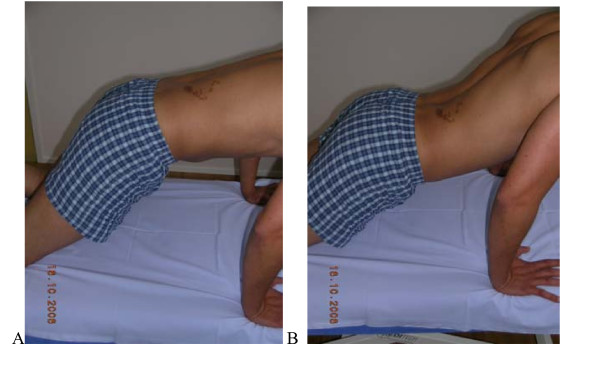
**Test protocol – Rocking forwards. A**. **Correct – **Rocking forwards without extension movement of the low back.**B Not correct **Hip movement leads to extension of the low back **Rating protocol: **As patients did not know the tests, only clear movement dysfunction was rated as "not correct". If the movement control improved by instruction and correction, it was considered that it did not infer a relevant movement dysfunction.

**Figure 7:**
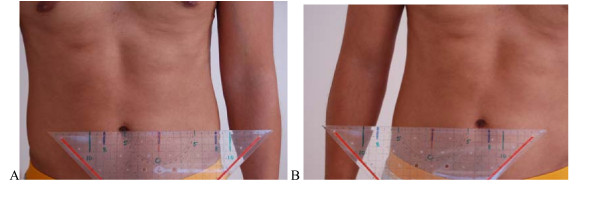
**Test protocol -One leg stance**. From normal standing to one leg stance: measurement of lateral movement of the belly button. (Position: feet one third of trochanter distance apart). **Correct – **The distance of the transfer is symmetrical right and left. Not more than 2 cm difference between sides. **B Not correct **Lateral transfer of belly button more than 10 cm. Difference between sides more than 2 cm. **Rating protocol: **As patients did not know the tests, only clear movement dysfunction was rated as ''not correct''. If the movement control improved by instruction and correction, it was considered that it did not infer a relevant movement dysfunction.

**Figure 8:**
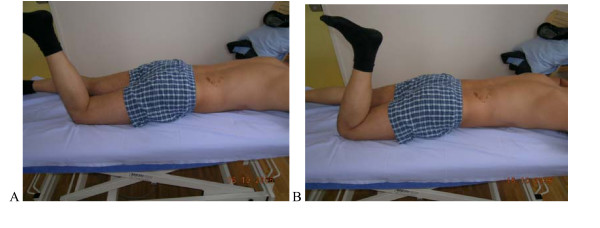
**Test protocol – Prone lying active knee flexion. A**. **Correct – **Prone lying active knee flexion at least 90° without (rot) movement of the low back and pelvis. **B Not correct **Pelvis rotates with knee flexion. **Rating protocol: **As patients did not know the tests, only clear movement dysfunction was rated as "not correct". If the movement control improved by instruction and correction, it was considered that it did not infer a relevant movement dysfunction.

**Figure 9:**
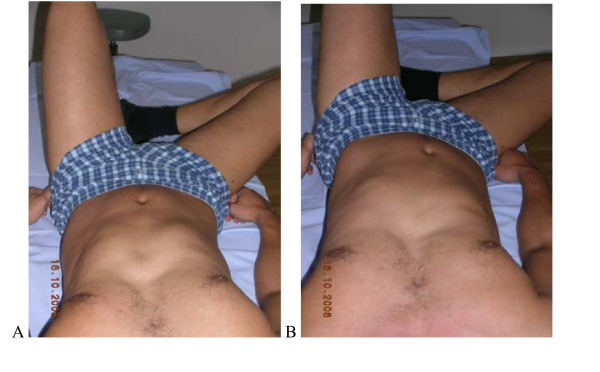
**Test protocol -Crook lying**. (supine, knees bent), **A**. **Correct – **Active abduction of the hip without rotational movement of the pelvis and low back. **B Not correct **Belly button moves sidewards, pelvis rotates or tilts. **Rating protocol: **As patients did not know the tests, only clear movement dysfunction was rated as "not correct". If the movement control improved by instruction and correction, it was considered that it did not infer a relevant movement dysfunction. **Rating protocol: **As patients did not know the tests, only clear movement dysfunction was rated as "not correct". If the movement control improved by instruction and correction, it was considered that it did not infer a relevant movement dysfunction.

We used ten active movement tests based on descriptions by Sahrmann [7] and O'Sullivan [9] [Figure 1, 2, 3, 4, 5,6, 7, 8, 9]. The test battery consisted of three tests for flexion and extension control and four tests for rotational control. To perform all of the tests, a patient needed approximately 10 minutes. The videos were all recorded within two weeks. The criteria for correct and incorrect performance are presented in Table 2.

### Analysis

The data were analysed by SPSS 14.0 for Windows [SPSS Inc. North Michigan Avenue, Chicago IL, 60611]. Rates of inter- and intra-observer agreement were analysed by calculating the percentage of agreement, determining the kappa coefficient between two pairs between all four raters. Confidence intervals [95%] were calculated for the values. A kappa coefficient of 1.0 indicates full agreement beyond chance. Values greater than 0.80 are generally considered excellent, values between 0.60 – 0.80 substantial, 0.4 – 0.6 good, 0.4 – 0.2 fair and values < 0.20 are poor [30].

We decided that a test should have kappa above 0.4 for both inter- and intratester value as well as the average of them. Further on, the lower bound of confidence interval [95%] should be over 0.2 being able to declare the reliability at least fair. To test whether the experience plays a role for the reliability, the scores of two experienced therapists were compared with the scores of two less experienced colleagues.

## Results

**Figure 10:**
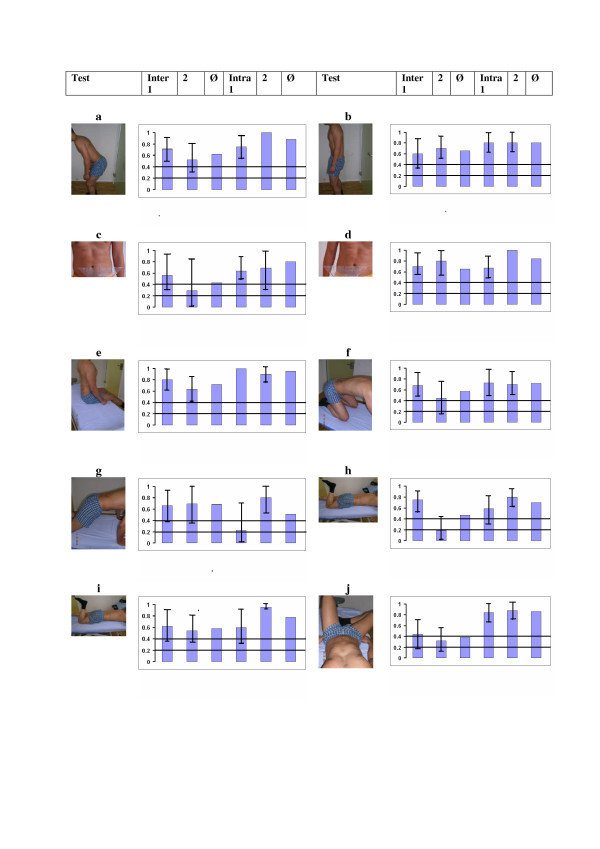
**Results overview**. The kappa values for inter-rater and intra-rater reliability per pair or person, confidence interval 95% and average value 4 a. Waiter bow; 4 b. Pelvic tilt; 4 c. One leg stance right; 4 d. One leg stance left; 4 e. Sitting knee extension; 4 f. Rocking backwards; 4 g. Rocking forwards; 4 h.Prone knee bend extension; 4i. Prone knee bend rotation; 4 j. Crook lying **: **Kappa values for: inter-rater first pair of raters (inter 1), second pair (2), average of pair 1 and 2 (Ø), intra-rater for the first rater (intra 1), for the second rater (2) and average of both (Ø). The kappa value should be over 0.4 to be good (bar) and the lower bound of confidence interval at least 0.2 to be fair (line).

Table 2. shows the attained values for the inter- and intrarater reliability and Figure 10. gives an overview of the Kappa values, 95% confidence intervals and mean values. Five out of ten tests showed a substantial inter-observer reliability [k > 0.6], four tests had Kappa values between 0.40 and 0.60 [good] and one test was under 0.4 [fair]. Lower values of the 95% CI were > 0.2 in 5 tests. The percentage agreement varied between 65% – 97.5%. All the results, except for two tests in the second pair of observers were highly significant [p < 0.01].

For the intra-observer reliability, five tests out of ten showed an excellent reliability [k > 0.80]. Four further tests had a substantial reliability [k = 0.6–0.8] and one was moderate [0.51]. All the results were significant at a *p *< 0.001 level [Table 2].

**Table 2:** Results of Inter- and intra-observer reliability

**Results inter-observer reliability**										
	***Waiters ***	***Pelvic***	***One leg***	***One leg***	***Sitting***	***Rocking***	***Rocking***	***Prone knee***	***Prone knee***	***Crook***
***test***	***bow***	***tilt***	***stance R.***	***stance L.***	***Knee ext.***	***Fx.***	***Ext.***	***Bend Ext.***	***bend Rot.***	***lying***

***pair 1***										
**Kappa Coefficient (CI 95%)**	0.71 (0.50–0.92)	0.60 (0.36–0.84)	0.56 (0.27–0.85)	0.70 (0.57–0.93)	0.80 (0.61–0.99)	0.68 (0.45–0.91)	0.66 (0.39–0.93)	0.75 (0.52–0.98)	0.62 (0.37–0.87)	0.44 (0.18–0.70)
**% Agreement**	85.7	80.0	88.0	88.0	90.4	88.0	92.8	97.6	90.5	78.6
**Std Error**	0.11	0.12	0.14	0.19	0.09	0.18	0.14	0.11	0.13	0.13
P-value	< 0.001	< 0.001	< 0.001	< 0.001	< 0.001	< 0.001	< 0.001	< 0.001	< 0.001	0.003
***pair 2***										
**Kappa (CI 95%)**	0.52 (0.29–0.75)	0.70 (0.48–0.92)	0.29 (0.00–0.84)	0.80 (0.54–1.00)	0.64 (0.40–0.88)	0.45 (0.16–0.74)	0.69 (0.37–1.00)	0.19 (0.00–0.42)	0.54 (0.28–0.80)	0.32 (0.10–0.54)
**% Agreement**	75.0	92.5	97.5	92.5	95.0	90.0	92.5	87.5	87.5	65.0
**Std Error**	0.12	0.11	0.26	0.13	0.12	0.15	0.17	0.12	0.14	0.11
**P-value**	< 0.001	< 0.001	0.053	< 0.001	< 0.001	0.004	< 0.001	0.11	0.001	0.012

**Kappa Ø**	0.62	0.65	0.43	0.65	0.72	0.57	0.68	0.47	0.58	0.38

**Results intra-observer reliability**										

***Observer 1***										
**kappa**	0.75 (0.55–0.95)	0.80 (0.61–0.99)	0.64 (0.40–0.88)	0.67 (0.44–0–90)	1.0	0.73 (0.44–1.00)	0.22 (0.00–0.64)	0.59 (0.29–0.81)	0.60 (0.31–0.91)	0.84 (0.66–1.00)
**% Agreement**	97.5	95.0	92.5	87.5	100	97.5	95.0	92.5	92.5	97.5
**Std Error**	0.10	0.01	0.12	0.12	0.0	0.15	0.24	0.15	0.15	0.09
**P-value**	< 0.001	< 0.001	< 0.001	< 0.001	< 0.001	< 0.001	0.16	< 0.001	< 0.001	< 0.001
***Observer 2***										
**Kappa (CI 95%)**	1.0	0.8 (0.61–0.99)	0.69 (0.29–0.99)	1.0	0.9 (0.77–1.00)	0.70 (0.48–0.92)	0.8 (0.56–1.00)	0.8 (0.62–0.98)	0.95 (0.94–0.96)	0.88 (0.71–1.00)
**% Agreement**	100	95	100	100	100	97.5	100	92.5	100	97.5
**Std Error**	0.0	0.09	0.23	0.0	0.07	0.11	0.12	0.94	0.05	0.09
**P-value**	0.000	< 0.001	< 0.001	0.000	< 0.001	< 0.001	< 0.001	< 0.001	< 0.001	< 0.001

**Kappa Ø**	0.88	0.80	0.67	0.84	0.95	0.72	0.51	0.70	0.78	0.86

The best inter-observer reliability was shown in four tests; posterior tilt, one leg stance left, sitting knee extension and extension test on four point kneeling [both pairs of raters k > 0.60]. The first observer pair, the more experienced ones, rated 8 out of 10 tests highly reliably [k = 0.60–0.80] and two further tests, the one leg stance right and crook lying abduction test, had moderate reliability [k = 0.56 and 0.44;]. The second observer pair rated four of the ten tests highly reliably [k = 0.64–0.80], three with moderate reliability [k = 0.45–0.52] and three [prone knee bend extension, one leg stance right and crook lying abduction] had fair reliability [k = 0.19–0.32].

The most reliable tests overall [for both rater pairs] were: pelvic tilt for extension dysfunction, one leg stance left for rotational dysfunction and the sitting hamstrings test for flexion dysfunction. The poorest test was abduction in crook lying for rotational dysfunction where both rater pairs showed low kappa values [k = 0.44; CI 0.18–0.70 and k = 0.32 CI 0.10–0.54]. Also prone knee bend and rocking forwards tests have to be questioned critical where one rater pair or intrarater results were low.

## Discussion

Inter- and intra-observer reliability of the majority of the MCD tests was good [k > 0.6]. In the intra-observer comparison, one of the two persons tested, could highly reliably [k = 0.69–1.0] rate all the tests. The second person tested rated 8 out of 10 tests [k = 0.60–1.0] highly reliably, one test moderately [k = 0.59; and one test fairly [k = 0.22].

It is worth commenting that all four therapists mentioned that better protocol training could have been carried out beforehand. There were two pairs of observers. The more experienced pair demonstrated a better inter-rater reliability than the less experienced pair, which is comparable with the findings by Dankaerts [16] and hypothesised by van Dillen [15] In the intra-observer reliability the less experienced person showed a higher reliability in the rating [all tests k > 0.69].

On average, the LBP patients were performing 3–4 tests incorrectly out of 10 and the healthy controls on average 1 test out of ten. We did not follow this data further in this study.

The findings of our study support the results of an earlier study, in which the reliability of the assessment of movement impairment items was found to be good. Van Dillen et al [15] used a whole package of physical examination items in order to categorize the patients in an impairment dysfunction subgroup. They found a very high agreement for symptom behaviour among the examiners [k > 0.89 and % agreement > 98%]. Further, they examined the reliability of observation of spinal alignment and movement items. In general the interpretation of the spinal alignment was slightly lower [k = 0.27–0.58] than for the observation of the active movements [k = 0.26–1.00]. In their examination package they used six similar tests as this study. A comparison of the kappa coefficients of these two studies is shown in Table 5. In general the results of the two studies are similar. Van Dillen et al. examined the flexion movement in standing by the relative flexibility which means the observer rates whether the low back moves earlier, easier or faster than the hip. This item is partly comparable with our test for flexion in standing known as the "waiters bow", which tests the ability of the subject to separate the movement of the hip and low back. Basically the underlying concept is similar, but, the test is performed and rated differently. A difference between our study and the study by van Dillen et al. was, that in the latter study therapists gained information from the patients regarding their symptoms during the test, making it easier to value the individual items, whereas in our study the raters were blinded to the patient's symptom responses and history. Van Dillen et al. commented that they trained the raters "fairly well" and they expressed doubt whether other examiners, not trained by the developer of their tests, could value the examinations as well. Our study confirms that it is possible to train the accurate analysis of movement, albeit with a slightly lower precision. This is important because clinicians rely on their assessment of movement dysfunction for inspection of movement. In contrast the reliability of many manual therapy and orthopaedic diagnostic methods has been shown to be poor [19-25].

We introduced a test, the "single- leg stance", which van Dillen et al. did not use. The basis of the test is that with an extension rotational dysfunction there can be marked differences in the lateral shift of the pelvis relative to the trunk [7]. We standardized this test according to Klein-Vogelbach [26] so that normal stance width would be one third of the distances between trochanters. A similar study has been performed on side to side weight bearing [27] which demonstrated a significant difference between patients with low back pain and healthy controls.

Another test which is frequently used in the clinic is the posterior tilt of pelvis for extension dysfunction [7]. This was one of the most reliable tests in our study [k = 0.6–0.8].

Dankaerts et al [16] reported an almost perfect agreement [k = 0.96 and percentage agreement 97 %] between two expert examiners rating a MCD classification. Thirteen further clinicians classified the same cases. Kappa-coefficients [mean .61 and range .47 -.80] indicated a substantial reliability. They stated that increased familiarity with the tests improved reliability. The difference between their study and ours is, that their 2 raters at first saw the whole subjective and whole physical examination of the patients and in the part 2, 13 clinicians had the written notes of the patients history and pain behaviour with video footage of functional movements. This is clinically most relevant. However, in a diagnostic sense, evidence based medicine demands reliability of individual tests protocols which we did in our study [28]. In the Dankaerts et al. study, no conclusions can be made about individual tests as the individual tests were not analysed in the classification process.

White & Thomas [29] investigated the reliability [N = 37] of sixteen tests of the Movement System Balance approach developed by Sahrmann. They found a satisfactory reliability between raters [table 3]. Five of these tests were also used in our study. The difference with our study is that they used provocation tests, meaning that when the tests caused pain and the correction of the faulty movement pattern relieved the pain, the test was rated positive. In our study, only the quality of the movement was rated as correct or incorrect. So to say, we rated only the visible information of the observation of the movements, which is supposed to be one of the key competencies of physiotherapy.

**Table 3:** Comparison of Kappa coefficients

Test	This study Ø Kappa	Van Dillen et al 1998	White & Thomas 2002	Murphy et al 2006
Sitting knee Extension	0.72	0.58	0.17	
Crook lying hip abduction	0.38	0.60	0.50	
Prone knee bend extension	0.47	0.76	0.22	
Prone hip extension				0.74
Prone knee bend rotation	0.58	0.43		
Rocking 4 point kneeling fx	0.57	0.78	0.62	
Rocking 4 point kneeling ext	0.68	0.51	0.39	
Fx standing relative flexibility		0.51	0.55	
Waiters bow	0.62			

Murphy et al [30] [N = 42] investigated one test, prone hip extension, that was rated positive if the lower back moved when the hip was extended. Inter-rater reliability was substantial with k = 0.72 for left and 0.76 for right hip. The difference to our study was that we only let subjects bend the knee and rated the test positive if the low back was moving.

MCD links closely to clinical instability. Cook [31] has established the pattern of clinical instability of the low back through a qualitative Delphi study. In his study, manual therapists [N = 168] thought that the physical findings of poor co-ordination, proprioception deficits and loss of control of the active movements were most important. According to Panjabi [32], the neural control system represents the control of movement and motor control.

The examination of the motor control of individual muscles is also linked to MCDs and reliability studies also have been carried out on examination of the passive lumbar movements. A good reliability has been demonstrated of the examination through ultrasound imaging compared to MRI of the primary stabilizing muscles, the transversus abdominis [33,34] and of the multifidus [35]. There is also some evidence to show at least a moderate reliability for palpation and a pressure biofeedback device of these muscles [36]. However, muscle diameter, muscle tests, movement tests, volitional movement – they all measure different aspects of motor function. EMG and kinematic studies might be more accurate for motor control assessment. It seems questionable, however, how readily these systems can be employed in daily private physiotherapy practice settings. The passive movements are clinically assessed through passive intervertebral movements and palpation. Many studies have shown that these tests are unreliable [19-25]. Therefore, the evaluation of active functions may be more rewarding. Obviously, more studies of reliability of the clinical examination without ultrasound and other costly devices are still needed.

This study does not say anything about the validity of these tests, i.e., how do patients with LBP perform these tests compared to subjects without back pain. Test-retest reliability of MCD should be examined as well and the normative values for correctly performed tests should be established in order to be able to proceed to outcome studies in this subgroup of patients with non specific low back pain.

## Conclusion

This study demonstrates that MCD tests of the lumbo-pelvic region have a good to substantial inter- and intrarater reliability The best all over reliability [k > 0.6] was shown in the "waiter's bow" and "sitting knee extension" test for flexion dysfunction, pelvic tilt for extension dysfunction as well as one leg stance difference for rotational dysfunction. In clinical settings it seems advisable that patients are rated by the same therapist as intra-observer reliability is better than inter-observer reliability.

## Competing interests

The author(s) declare that they have no competing interests.

## Authors' contributions

HL acquisited the data, made the videos, calculated statistics and was the main writer of the paper. JK was involved in the planning, methodological considerations, analysis of the data and revising the paper. EdB was involved in the planning, methodological considerations and revising the paper. OA was involved in the planning, methodological considerations and revising the paper. All authors read and approved the final manuscript.

## Note

Table 2-Results of the inter- and intra- observer reliability

## Pre-publication history

The pre-publication history for this paper can be accessed here:


